# Learner experiences of a carer-focused massive open online course on psychosis: A qualitative study

**DOI:** 10.1177/20552076261470199

**Published:** 2026-08-25

**Authors:** Kalya Win Aung, Angela Kibia, Juliana Onwumere

**Affiliations:** 1 Department of Psychology, Institute of Psychology, Psychiatry and Neuroscience, King’s College London, London, UK

**Keywords:** carers, families, psychosis, schizophrenia, digital interventions, massive open online course

## Abstract

**Background:**

People supporting individuals with psychotic disorders play a vital role in community-based mental health care but often face their own health and wellbeing challenges and limited access to support. Digital interventions, including online courses, may help address these unmet needs.

**Purpose:**

This study explored participants’ experiences of the Caring for People with Psychosis and Schizophrenia MOOC, using the Kirkpatrick Evaluation Model, a widely applied framework for evaluating educational programmes.

**Methods:**

Individual semi-structured interviews were conducted online with participants who accessed the course. Interviews were audio-recorded, transcribed verbatim, and analysed using reflexive thematic analysis. Findings were then mapped onto the four levels of the Kirkpatrick model to explore learner reactions, knowledge gains, behavioural change, and wider impacts of the MOOC.

**Results:**

Nine participants (six carers and three non-carers) were interviewed, and five key themes were identified. Learners described enrolling during periods of emotional difficulty and uncertainty and found the course validating, informative, and connecting (reaction). They reported greater understanding of psychosis, caregiving strategies, and self-care approaches (learning), which increased their sense of confidence. Many carers applied this knowledge in their daily routines, communication, and relationships (behaviour), reportedly improving perceived wellbeing for themselves and their relatives (impact). The MOOC’s flexible and multimodal design supported meaningful engagement alongside other responsibilities.

**Conclusions:**

Preliminary evidence from this model of online training suggests that a globally accessible MOOC can connect learners, enhance understanding, and build confidence in caregiving for psychosis. Subjective reports indicate the potential of MOOCs to address learners’ informational and emotional needs through flexible, supportive online education.

## Introduction

Psychotic disorders, including in their most common form, schizophrenia, are severe mental health conditions typically characterised by marked disruptions in a person’s perception, thinking processes, affect, and behaviour. According to the Global Burden of Disease 2019 study, an estimated 24 million people worldwide are living with schizophrenia, with approximately 1.3 million new cases each year.^1,2^ Psychotic conditions will typically follow a relapsing-remitting course,^3^ and more than two-thirds of affected individuals continue to experience symptoms for at least a decade after their first onset.^4^ As a result, psychotic disorders are expected to generate substantial ongoing care needs, increasing demands on health and social care systems.

### Support for people living with psychosis

The term ‘informal carers’ refers to relatives, significant others, and friends who provide unpaid support to people living with psychosis. Evidence attests they play a central role in supporting people with psychosis.^5^

Unpaid carers often provide the equivalent of a full working week of support for people with schizophrenia, with around one in five delivering 50 hours or more,^6^ highlighting their critical role in mental health care provision.^7^ However, carers of people with psychotic disorders can experience high levels of psychological distress, including depression,^8^ anxiety,^9^ trauma-related responses,^10,11^ sleep disturbances,^12,13^ and burnout and fatigue.^14,15^ Levels of poorer psychological health among carers of individuals with psychosis exceed those reported by their non-carer peers^16^ and by carers of individuals with other long-term mental^17^ or physical health conditions.^18^

The impacts of the carer role can also be registered in terms of carers’ reports of financial strain,^19,20^ disrupted routines,^21^ and reduced time for personal and social activities.^19,22^ These caregiving challenges can be enduring, with peak levels particularly during early illness phases and periods of crisis or relapse (e.g., following hospital admission).^9,23^ These can often be times when carers are exposed to illness-related situations for the first time, and their understanding of events, access to relevant information, resources, and support are limited.^24–27^

### Digital innovations for carers

Despite their crucial role, evidence attests that many carers in psychosis have unmet needs, including access to clear information about the illness, practical advice on navigating the caring relationship, and support.^19,20,28^ While clinical and best practice guidelines across the globe recommend psychoeducation and family support interventions,^29,30^ implementation can be limited. Many carers face practical barriers, such as travel distance and scheduling conflicts,^31^ as well as structural inequalities within services.^32^ For example, in the UK, only 12% of patients with family contact are offered family interventions, including psychoeducation,^33^ with uptake particularly low among some ethnic minority groups compared with White British peers.^34^

Digital innovations are increasingly being explored to deliver information and support interventions in psychosis, offering a promising way to mitigate barriers to access and support recovery for both people living with psychosis^35^ and their carers.^26,36,37^ Technologies, such as mobile health apps, telepsychiatry, web-based psychoeducation, and online peer support forums have demonstrated potential to improve carer wellbeing,^26,36,38,39^ increase access to mental health services,^26,35,36,38,39^ and are generally perceived as feasible to implement and acceptable to carers.^26,36,38,39^ Within this landscape, massive open online courses (MOOCs) have emerged as scalable, low-cost, and accessible digital learning tools that can reach diverse learners, including those traditionally underserved by formal education systems.^40^ However, access is influenced by factors such as digital literacy, language, internet connectivity, and available time. As global internet usage continues to grow, reaching approximately 68% of the world population and 93% in high-income countries in 2024,^41^ more unpaid carers are seeking support and resources online.^42^ MOOCs therefore represent a particularly promising avenue to deliver evidence-based psychoeducation at scale, although efforts to address digital exclusion remain important to maximise equitable access.

### Massive open online courses

MOOCs are typically defined by four key features comprising their: (i) ability to enrol large numbers of learners (“massive”); (ii) wide unrestricted access without formal prerequisites (“open”); (iii) delivery via web-based platforms (“online”), and (iv) structured curriculum guided by specific learning objectives (“course”).^43^ These features render MOOCs a practical way to support learners who might struggle to access traditional services due to time, cost, location, or lack of tailored information.^44–46^

While MOOCs have shown early promise in improving knowledge and caring skills for carers of people with dementia and other chronic conditions,^47^ their potential for addressing caregiving needs in psychotic disorders is less clear. Compared with other conditions, psychosis presents distinct caregiving demands, yet little is known about how learners experience psychosis-specific MOOCs, which design features of these courses are most meaningful, and whether participation is perceived to influence outcomes beyond knowledge acquisition. Understanding these experiences is essential to ensure interventions are accessible, relevant, and supportive.

Psychoeducational interventions for carers may operate not only by increasing illness-related knowledge, but also by supporting reflective and metacognitive processes. In the context of psychosis, metacognition has been defined as the capacity to monitor, understand, and modulate one’s own and others’ mental states, and impaired metacognitive functioning has been linked to symptom severity, insight, and functional outcomes.^48^ For carers, improved understanding of psychosis may therefore support more reflective responses to distressing experiences, enhance confidence, and reduce self-blame.

This study, therefore, sought to qualitatively examine learners’ experiences of engaging with a MOOC focused on caregiving in psychosis, guided by the Kirkpatrick Evaluation Model to explore reactions, learning, behaviour, and perceived impact. Although the MOOC was designed primarily for informal carers, it is openly accessible and may also attract professionals, students, and individuals with a broader personal or educational interest in psychosis. The study also explored factors related to accessibility and considered implications for future digital interventions.

## Methodology

### Study design

This study employed a cross-sectional design using individual semi-structured interviews to explore learner experiences of participating in a MOOC. This report follows the Consolidated Criteria for Reporting Qualitative Research (COREQ) guidelines (Supplementary File 1).^49^

The interview schedule was developed with reference to the Kirkpatrick Evaluation Model, an established framework for assessing the effectiveness of training programs.^50,51^ Kirkpatrick’s model comprises four levels: (1) Reaction – learners’ satisfaction and initial responses to the course; (2) Learning – knowledge and skills acquired; (3) Behaviour – self-reported behavioural application of learning in caring contexts; and (4) Impact – the perceived broader personal and professional effects of participation.

In addition to the Kirkpatrick framework, the interview guide was iteratively developed by the research team to ensure relevance and clarity, drawing on existing literature on caregiving and digital learning as well as the study aims.^52,53^ Questions were piloted informally within the research team and refined to encourage in-depth responses. The guide was used flexibly, allowing the interviewer to follow participants’ experiences and appraisals.

Each of the Kirkpatrick levels was explored through open-ended questions aimed at understanding the participants’ experiences with the MOOC and its outcomes.

• Level 1: Reaction. Questions focused on participants’ immediate responses to the course, including satisfaction with the content, delivery, and their individual motivations for engaging with the MOOC. It aimed to capture initial impressions and reactions.
• Level 2: Learning. Questions focused on exploring participants’ knowledge and insights gained from the course. It asked participants to identify content that was most helpful and any content gaps. It also assessed if and how the course enhanced their understanding of caring for individuals with psychosis and schizophrenia.
• Level 3: Behaviour. Questions focused on how participants applied course concepts in real-world settings, including any self-reported behavioural changes in caring approaches and confidence in addressing the needs of individuals with psychosis and schizophrenia.
• Level 4: Impact. Questions explored the perceived broader effects of the course on participants’ lives, including positive outcomes, improvements in personal or professional areas, and any challenges faced during participation.

The full interview guide can be found in Supplementary File 2.

### Caring for people with psychosis and schizophrenia massive open online course

The Caring for People with Psychosis and Schizophrenia MOOC is a global online course designed to support informal carers of individuals experiencing psychosis, including schizophrenia, and any other stakeholders with an interest in the area. It was developed by a multidisciplinary team at King’s College London, including psychiatrists, psychologists, pharmacists, nurses, and individuals with lived experience of psychosis and caregiving.^54^ The course draws on both clinical expertise and personal narratives to provide learners with practical knowledge and insight into caring for someone with psychosis.

The MOOC is delivered for free via FutureLearn, a digital learning platform that hosts short courses created by universities and professional organisations worldwide (https://www.futurelearn.com/courses/caring-psychosis-schizophrenia). It runs over four weeks, with a study commitment of approximately three hours per week.

The course adopts a flexible and accessible format, combining video lectures, readings, and peer discussion forums to support diverse learning needs and promote learner interaction. It is openly available without prerequisites, making it accessible to carers, professionals, and members of the public. The MOOC covers key areas including understanding symptoms of psychosis such as hallucinations, delusions, and negative symptoms, communication strategies, medication, physical health, and substance use, as well as carers’ wellbeing, recovery-oriented approaches, family impacts, and practical strategies for caregiving.

### Participants

Eligible participants were: (1) adults aged 18+ years and (2) a registered learner on the Caring for People with Psychosis and Schizophrenia MOOC, offered by King’s College London via the FutureLearn platform, between May 2024 and April 2025.

No formal inclusion or exclusion criteria were applied regarding the extent of course completion, which was self-reported by participants. Participants were eligible if they had enrolled on the course and were willing to reflect on their experiences. This approach was taken to reflect real-world patterns of MOOC engagement, where learners often vary in the extent to which they complete course material,^47^ and to capture a broader range of user experiences, including partial participation.

Recruitment was conducted directly through the course platform and may therefore have resulted in a sample of more engaged or positively inclined participants. A link to an online information sheet and consent form, hosted on Qualtrics, was embedded within the course modules. Learners who expressed interest in providing course feedback were directed to a webpage containing detailed information about the voluntary nature of the study, data handling procedures, and their rights as participants.

Those providing consent were asked to share basic demographic information (e.g., gender, age, country of residence, and caregiving experience). Participants were not required to have direct caregiving experience. Individuals without caring roles were included as they represented a broader group of learners engaging with the MOOC (e.g., those with professional or personal interest in psychosis). Because the sample included both carers and non-carers, analysis considered whether themes were shared across learner groups or primarily grounded in carer accounts. Where relevant, differences between carer and non-carer perspectives are highlighted in the Results. Participants were also invited to indicate their relationship to the person they had experience supporting. Those who completed this form were contacted by email to arrange a one-to-one interview.

Sample size was guided by the principle of information power,^55^ which emphasises the richness and relevance of data rather than aiming for a pre-determined number of participants. The approach recognises that relatively smaller samples can be sufficient when the study aim is narrow and participants have direct and relevant experience of the topic area. Given the focused aim of exploring experiences of a single MOOC, and the specificity and depth of the interview material, this sample was considered sufficient to support meaningful analysis. Approximately 12 interviews were initially anticipated based on feasibility and pragmatic considerations, but the final sample size was not fixed in advance. Sampling instead proceeded iteratively with ongoing assessment of information power during data collection and analysis.

Final sample adequacy was judged through consideration of the depth, relevance, and richness of the data in relation to the study aims, alongside the quality of the dataset and consistency of emerging themes across participants. Although participants without direct caregiving experience were included, their perspectives contributed to understanding the broader applicability of the MOOC. Diversity in age, gender, caregiving role, and geographic location further enhanced the contextual richness of the dataset.

The study did not involve formal patient and public involvement in the design or conduct of the research. However, the MOOC itself was developed with considerable patient and public involvement and engagement from individuals with lived experience of psychosis and caregiving and mental health professionals.

### Data collection

Data were generated through individual semi-structured interviews conducted remotely between May 2024 and April 2025, using the Microsoft Teams video conferencing platform. All interviews were carried out by the first author (KWA), a female researcher experienced in qualitative research on sensitive topics, who had no prior relationship with participants. Participants were informed of the interviewer’s role as a research assistant, the goals of the research, the voluntary nature of participation, and their right to withdraw at any time. They were also informed that the study was independent of course assessment and that both positive and critical reflections were equally valued. The interviewer adopted a neutral and non-judgemental style to minimise social desirability bias and encourage open discussion of experiences. Verbal consent was obtained at the start of each interview, including consent for audio recording and subsequent verbatim transcription.

Follow-up questions were asked throughout the interviews to clarify or expand on participants’ responses. At the conclusion of each interview, participants were also invited to share any final thoughts regarding their experience with the MOOC. Interviews were scheduled for up to 60 mins. Participants were compensated with a £15 voucher for their time.

### Data analysis

All interviews were transcribed verbatim, and participants were pseudonymised using anonymity codes. Data were analysed using reflexive thematic analysis, following Braun and Clarke’s six-phase process,^56–58^ and reported in accordance with the Reflexive Thematic Analysis Reporting Guidelines (RTARG).^58^ We adopted a hybrid analytic approach that combined inductive and deductive elements. Codes and themes were grounded in participants’ accounts, with deductive structuring guided by the Kirkpatrick Evaluation Model,^51^ a framework used in previous evaluations of MOOCs and other digital learning interventions.^59–61^ This approach is consistent with reflexive thematic analysis, which allows themes to be generated from the data while also using theory to guide interpretation. Applying the framework after initial coding helped ensure that analysis remained grounded in participants’ accounts and was not constrained by predefined categories.

The analysis began with the first two authors (KWA and AK) familiarising themselves with the interview transcripts and generating initial codes in NVivo (version 15; QSR International). KWA brought contextual sensitivity from conducting the interviews, while second author, AK, offered an external perspective. Regular meetings were held to facilitate analytic dialogue and collaborative interpretation, with discussions used to explore different readings of the data and deepen thematic development, consistent with a reflexive approach.^62^ Throughout the analytic process, the researchers maintained analytic memos to document evolving interpretations and points of divergence in coding. Iterative coding logs were also used to track changes in code development. These materials informed ongoing discussions between KWA and AK, supporting reflexive engagement with the data and refinement of candidate themes. This inductive theme development was further supported through working tables that mapped codes to emerging thematic structures.

Themes were further discussed with the third author (JO) and mapped onto the four levels of the Kirkpatrick framework: Reaction, Learning, Behaviour, and Impact. This deductive mapping was carried out after inductive theme generation to ensure themes were grounded in the data before applying the evaluative framework. Final themes were developed collaboratively and shaped by the research team’s interpretive engagement with the data, as well as their critical reflection on positionality and assumptions. The research team comprised individuals with postgraduate-level education and diverse ethnic backgrounds, with expertise in mental health, carer research, and digital learning, which informed the analytic lens.

As the team were based at King’s College London and involved in developing the MOOC, we considered how this may have influenced the study, including recruitment through existing networks that could have led to more positive accounts, and the possibility that participants may have softened critical feedback due to this connection. We also reflected on how this insider position might have shaped interpretation during coding and theme development. To reduce these risks, the interviewer was not involved in course delivery or assessment and made clear during recruitment and interviews that the study was independent of the course. Regular team discussions were used to question interpretations and consider alternative explanations in the data. Participants were not invited to review transcripts or provide feedback on the findings but were offered a summary of the results upon study completion.

## Results

Participant interviews lasted between 20 and 60 minutes and the sample comprised nine participants (see Table 1 for a summary of participant characteristics and labels used for quotations). Five participants identified as female, three as male, and one as non-binary. Participants ranged in age from 18 to over 66+ years, with most falling between 46 and 65 years. Participants were based in the UK, Uganda, Oman, Greece, and Ghana. Six participants reported having current or previous caregiving roles, supporting either a child or a friend with a mental health condition. The remaining three participants had no direct caregiving experience and engaged with the MOOC due to personal or professional interest in psychosis. Seven participants self-reported completing the full four-week course (including one participant who repeated the course for a second time), while two participants self-reported not completing the course. Engagement primarily involved viewing video lectures and reading course materials, with fewer participants reporting participation in discussion forums. Their demographics are reported in aggregate to protect anonymity.

**Table 1.** Participant characteristics and quotation labels.

ID	Gender	Age group	Caregiving experience	Relationship	Living arrangement	Country	Completed course	Quotation label
001	Female	66+	Yes	Child	Not living together	UK	Yes	001_C_UK
002	Female	46–55	Yes	Child	Not living together	UK	Yes	002_C_UK
003	Female	46–55	No	—	—	Uganda	Yes	003_NC_UG
004	Female	56–65	Yes	Child	Not living together	UK	Yes (2^nd^ time)	004_C_UK
005	Male	56–65	Yes	Child	Living together	Oman	No	005_C_OM
006	Non-binary	18–25	No	—	—	UK	Yes	006_NC_UK
007	Female	56–65	Yes	Child	Not living together	Greece	No	007_C_GR
008	Male	36–45	No	—	—	Ghana	Yes	008_NC_GH
009	Male	66+	Yes	Friend	Not living together	UK	Yes	009_C_UK

**Figure 1.**
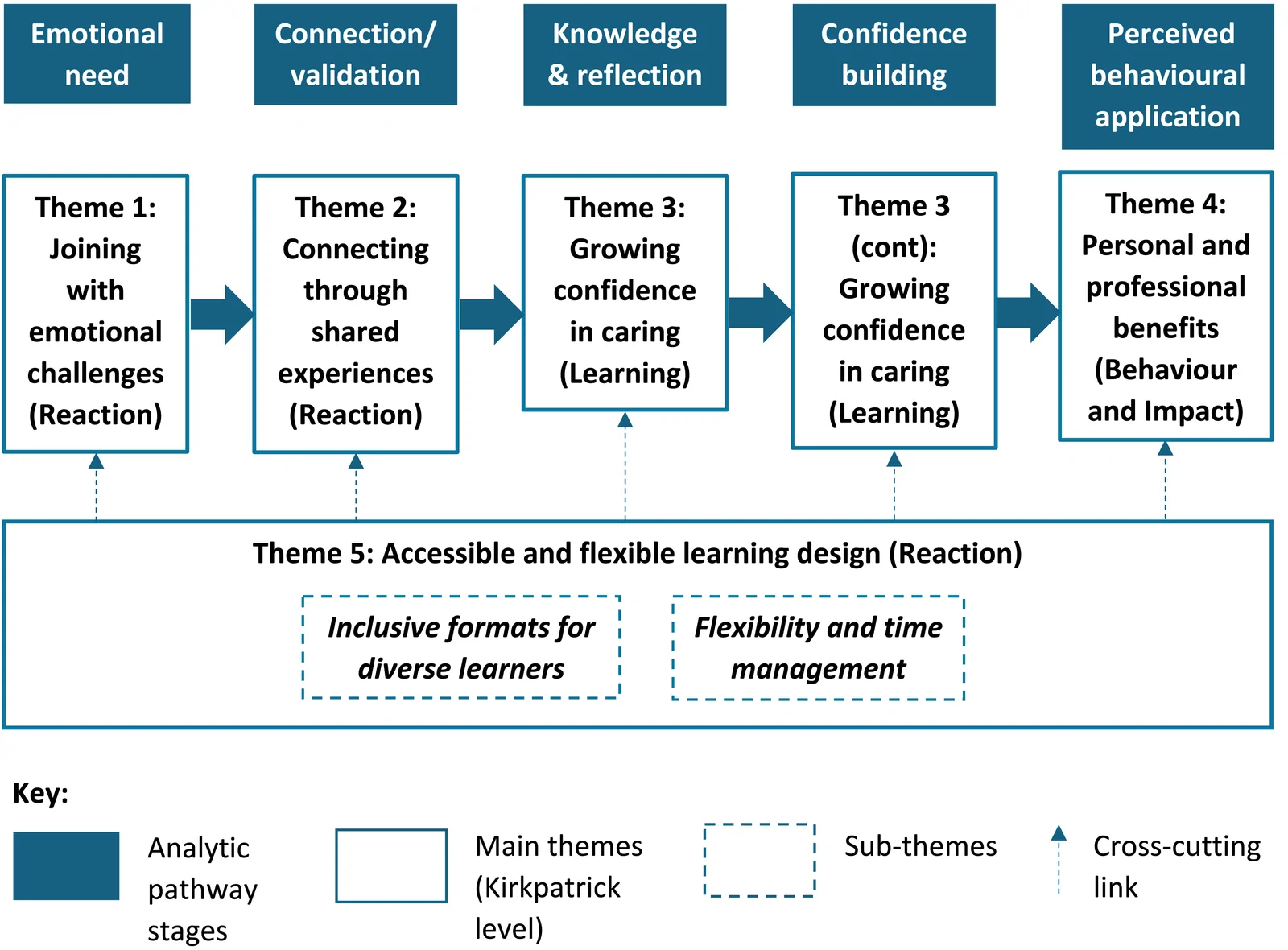
Analytic pathway of learner experiences and outcomes mapped to qualitative themes.

The reflexive thematic analysis yielded five themes. Themes were mapped onto the Kirkpatrick Evaluation Model to reflect participants’ reactions to the course (Themes 1, 2 and 5), perceived learning and confidence development (Theme 3), and reported behavioural and broader personal or professional impacts (Theme 4). Figure 1 presents the themes as a proposed pathway of engagement and change, illustrating how participants moved from emotional need and uncertainty towards greater understanding, confidence, and perceived application of learning. Table 2 provides a summary of each theme, its alignment with the Kirkpatrick framework, participant groups represented, key processes, and example outcomes. Theme descriptions with illustrative participant quotes are reported below.

**Table 2.** Summary of themes, Kirkpatrick levels, participant groups, key processes, and outcomes.

Theme	Kirkpatrick level	Participant group	Key process	Example outcome
Joining with emotional challenges	Reaction	Carers	Seeking information and support during uncertainty	Greater understanding
Connecting through shared experiences	Reaction	Mainly carers	Validation, normalisation, shared learning	Reduced isolation and greater sense of community
Growing confidence in caring	Learning	Mainly carers	Knowledge acquisition, reflection, self-awareness	Increased confidence, self-compassion, advocacy
Personal and professional benefits	Behaviour and Impact	Carers and non-carers	Application of learning in caring and professional contexts	Changes in caregiving approaches and career development
Accessible and flexible learning design	Reaction (cross-cutting)	Carers and non-carers	Flexible and inclusive engagement	Improved accessibility and participation

### Theme 1: Joining with emotional challenges (reaction)

This theme captures how carer participants began the MOOC during periods of uncertainty and emotional strain, seeking understanding and support in relation to psychosis.

Many carers reported starting the MOOC at a time when they felt lost and alone. These participants described experiencing social isolation, uncertainty about psychosis, direct and indirect consequences for the care recipient, and having limited access to reliable information about psychosis and schizophrenia.

Participants with caring roles recounted feeling unprepared for the onset of psychotic symptoms in their relative and often came across the course incidentally while searching more broadly for guidance, understanding, or reassurance. Some were referred to the course by support networks or healthcare professionals.

*“I didn’t know exactly what was going on. Suddenly something went wrong, and the hallucination happened… I was shocked. So that is why I came to study the course and to know exactly scientifically what he got.”* (005_C_OM).

*“I’ve come into the course because [my son] has encountered prejudice, violence, bias and I have encountered no knowledge to help him.”* (007_C_GR).

Carer participants reflected on the emotional complexity of a caring role and experiencing a rollercoaster of negative emotions that included guilt, self-doubt, and hopelessness, and an eagerness to make sense of their role in their relative’s experiences:

*“I’ve always felt as a parent that I try and search for things I could have done differently… it’s just difficult emotionally… I feel on the surface that I’m not really to blame because it was an illness.”* (002_C_UK).

*“A lot of times with the likes of schizophrenia and the way it’s portrayed… it’s almost like a no-hope illness.”* (001_C_UK).

Their emotional challenges were often compounded by a perceived lack of support from health care services. They described feeling ignored, excluded by professionals from decision-making processes about the care recipient, marginalised from care systems, and unsupported in ways that accounted for their cultural background or individual circumstances:

*“Carers feel very let down by the system… We need to really make sure specific and correct support is available for carers – culturally appropriate support.”* (004_C_UK).

*“[My friend] calls the emergency services regularly and [is] not getting any cooperation from his mental health team or his doctor.”* (009_C_UK).

### Theme 2: Connecting through shared experiences (reaction)

This theme captures how the MOOC enabled participants to connect with others’ experiences, helping to reduce isolation and foster a sense of shared understanding. These experiences of connection and normalisation were primarily emotional and relational in nature, helping participants feel less alone and more understood.

Participants described how the course brought together learners from diverse backgrounds while centring their caring experiences. Course elements such as discussion forums and video accounts from real carers and people with lived experience of psychosis encouraged feedback and dialogue between course learners.

For both carer and non-carer groups, the discussion forums offered a valued space to share reflections and learn from the experiences of others, which, in turn, helped to normalise some of their own feelings and situations and counter feelings of self-doubt, uncertainty, and isolation. Whether participants posted comments or chose to read the comments and dialogue exchange of others, both modes of engagement helped build a sense of belonging and community:

*“I thought the discussion boards were really useful to be able to… share ideas and have that sense of community.”* (006_NC_UK).

*“I like it that we can be interactive if we want… I also like reading their replies.”* (007_C_GR).

*“When some of the other learners made comments, it got me thinking… I may have experienced what the learner was talking about.”* (004_C_UK).

Participants described the video content featuring real carers and lived experience individuals as a powerful and relatable aspect of the course. For carers in particular, these stories helped reduce their feelings of isolation and make sense of their caring journeys:

*“Those that understand it… with the lived experience is excellent and it made me feel better because it meant [it] made you feel not alone.”* (001_C_UK).

*“The videos of the parents… that was really helpful. It was reassuring to see what they’ve been through and how they cope… On a personal level, it's helped me sort of think through difficult sort of things, because it's been very hard to come to terms with everything that's gone on.”* (002_C_UK).

However, some participants felt the discussion forums provided limited reassurance due to the lack of visible feedback of peer contributions:

*“There’s no review or feedback on the comments… so you don’t know whether the people are making sense, [or if] they are contributing effectively.”* (008_NC_GH).

Others felt limited by the asynchronous nature of the discussion forums and comment sections and offered their own solutions:

*“I logged on for the course early in the beginning, I didn’t have like many comments, but when I came back to the material later, there were comments and reactions and responses and likes… The interaction aspects are very important.”* (003_NC_UG).

*“I do hope … to discuss things live [with people on the course] … a roundtable discussion*.*”* (005_C_OM)

### Theme 3: Growing confidence in caring (learning)

This theme captures primarily carer participants’ perceived reports of feeling more confident in their caring role after enrolling on the course, through improved self-awareness, and greater recognition of their own support needs. In contrast to Theme 2, which focuses on relational understanding and reduced isolation, Theme 3 reflects internalised changes in self-efficacy, with carers describing a shift towards applying insight within their caregiving role. Some non-carer participants also described increased confidence in understanding and responding to psychosis-related experiences.

Many carers found the information on the course reassuring, as it provided confirmation they were on the right track in their coping and engagement approaches, and was perceived to enable them to advocate for their relative more effectively:

*“It said a lot about encouraging, emphasising good things… It sort of confirmed that that’s a good thing to do, and it reminded me that that’s a good way to carry on… I feel that I’m properly equipped.”* (002_C_UK).

*“I can probably stand up better for his rights. The course has helped me put everything into an order and it’s helped me make my decisions, which I will take after the course of how to help my kid.”* (007_C_GR).

Both carers and non-carers described becoming more aware of their own emotional and support needs. The course helped them recognise the importance of attending to their own wellbeing, being more intentional and proactive in seeking support for themselves. Some carers also reflected on preserving a sense of their own identity alongside their caring role:

*“Being confident to speak that I need support as well, it’s not only about the service user… sometimes as a carer you can lose your identity.”* (004_C_UK).

*“I hold a lot more compassion for myself… the course provided me with a lot of, like, reassurance, and that things won’t necessarily ever be the same again, but you’ll find your new normal and you’ll find ways to cope with it.”* (006_NC_UK)

For other carers, taking part in the course was linked to a shift in mindset towards having a more positive outlook with regards to the illness course, their relative, and in their own coping abilities:

*“The course… gave me more strength.”* (005_C_OM).

*“If you’ve got schizophrenia… there is hope… there should be a way of getting them hopefully back on their own feet.”* (001_C_UK).

Overall, confidence appeared to develop through both cognitive learning from course content and emotional normalisation arising from shared experiences.

### Theme 4: Personal and professional benefits (behaviour and impact)

This theme captures how participants applied course learning in practical ways, with participants describing perceived benefits for their caregiving and, in some cases, informing professional development or future aspirations.

Many carer participants described using knowledge acquired from the course to make changes in their caring behaviours and attitudes, which participants perceived as yielding direct benefits for their relative, their relationship and, for some, their career options:

*“I am applying everything as I can… I am taking him out to visit friends, to walk, to write, not to sleep all day… Before he was not talking to the people. Now talking to the people and contacting the people more better than before… he can do now everything.”* (005_C_OM).

*“It’s helped me to engage a bit different, not be as stressed about it. He is hearing a voice, but if I’m not stressed and ignore it and walk away or whatever… that has helped.”* (001_C_UK).

*“From the course I was able to see that one of the side effects of some of the drugs is weight gain… I can now say to him, apart from the fact you’re hardly getting any exercise, it could be a side effect of your drugs.”* (009_C_UK).

Both carers and non-carers said the course supported their professional development, either by informing their current roles or inspiring future career goals in mental health and caring:

*“Now I get this experience… I’m seriously thinking of applying to be an independent mental health advocate.”* (009_C_UK).

*“I see it [the course] as a stepping stone… paving the way for me to get that certificate I want in caregiving.”* (008_NC_GH).

*“If I then work with people with psychosis going forwards in my career, I have a bit more of an insight in how to support them as a professional and how to suggest support to their carers.”* (006_NC_UK).

Overall, participants applied the course in different ways. Carer participants primarily described changes in caregiving approaches and family interactions, while non-carer participants more frequently reflected on professional development, future career aspirations, and broader understanding of psychosis.

### Theme 5: Accessible and flexible learning design (reaction)

This theme, comprising two subthemes, captured participants’ reflections on the course as both accessible and flexible, enabling engagement that suited their needs, preferences, and daily demands.

#### Subtheme 5.1: Inclusive formats for diverse learners

Both carer and non-carer participants noted that the use of varied content formats, such as videos, transcripts, written materials, and discussion forums, helped support different learning styles and people with accessibility needs to engage with the course more easily:

*“If it was all just words, that would get a bit monotonous. The videos help to break it up… I learn a lot from seeing and hearing rather than reading.”* (009_C_UK).

*“I enjoyed the videos because again, with my learning challenges being dyslexic, I prefer to hear and see things… rather than always have to read.”* (004_C_UK).

*“I’m autistic and I struggle with auditory processing, so being able to then read the words [in the video transcript] was really, really useful.”* (006_NC_UK).

Participants also appreciated being able to revisit the course materials at their own pace and share them with others without any barriers, such as cost:

*“All the articles were… Open Access… I love the accessibility.”* (003_NC_UG).

#### Subtheme 5.2: Flexibility and time

Carers and non-carers welcomed the self-paced structure of the course, which enabled them to fit the course around busy daily routines, including work, family commitments, and sometimes care responsibilities. This flexibility made participation possible even amidst demanding and unpredictable schedules:

*“The course is very flexible and I’m able to read at work, at home… I read the course during my lunch time and sometimes when I get to work early… ample time is given to you.”* (008_NC_GH).

*“It wasn’t too long. It was manageable.”* (002_C_UK).

However, for some, the four-week duration felt too compressed for the volume of content, and some participants would have preferred a longer timeframe to learn and better absorb the material:

*“Time kinda ran away with me… I probably would suggest maybe making it more than four weeks… not necessarily increase the content, but… give it an extra week.”* (004_C_UK).

*“There was a bit of a challenge… it was a lot of information to try and take on board within a specific time frame… I needed a little bit of extra time to sift through it.”* (006_NC_UK).

## Discussion

This study explored learners’ experiences of participation in a MOOC focused on addressing needs of unpaid carers of people living with psychotic disorders. Data gathered from nine individual interviews suggested the MOOC offered an accessible, flexible, and supportive learning platform for both non-carers and carers – a group often overlooked by health systems despite their vital role in community-based mental health care and the management of psychotic disorders.^26,63,64^

Across participants’ accounts, the course was experienced as more than an information-delivery tool. Its value for carers and non-carers also lay in providing validation, peer support, and flexible access that enabled engagement in different ways. Participants also described that the course legitimised attention to their own wellbeing, suggesting that its impact extended beyond knowledge acquisition to include emotional and relational support.

These experiences are considered through the lens of the Kirkpatrick Evaluation Model,^51^ which is used to structure the remainder of this Discussion, focusing on participants’ reactions, learning, behavioural application, and perceived broader impacts of participation. Our results provide preliminary evidence that a MOOC may support knowledge acquisition, emotional well-being, and confidence in caring roles, and professional development.

### Impact of the MOOC on participants’ reactions (level 1)

Level 1 of the Kirkpatrick model focuses on participants’ immediate reactions to the educational intervention. Consistent with the growing trend of carers seeking health-related guidance online,^42,65,66^ many participants enrolled on the MOOC to gain reliable information on understanding and managing psychosis. Carers frequently described feeling emotionally overwhelmed, socially isolated, and unprepared for their relative’s illness. These experiences align with extensive evidence documenting carers’ unmet needs for illness information and emotional support,^19,20,28^ as well as research indicating that carers of individuals with psychosis can be at elevated risk of poor mental health compared with non-carers^16^ and carers of other long-term conditions.^17,67^ These challenges are often compounded for carers from ethnically and racially minoritised backgrounds^32,68^ or underserved groups^69–71^ who may experience additional systemic barriers. Although we did not collect detailed demographic information to determine whether our participants belonged to these groups, some narratives of exclusion from care decisions, discrimination, and culturally lacking support suggest that similar barriers may have been present in our data.

The MOOC’s self-paced, flexible structure supported integration with participants’ caring responsibilities or other roles, including work commitments. This reflects adult learning principles that emphasise the importance of self-directed learning for increasing motivation.^72,73^ Lived-experience videos featuring real carers, combined with learner-to-learner interactions in the discussion forums, provided emotional validation by normalising and contextualising shared experiences of carer participants.

Discussion forum interactions were reported to reduce feelings of isolation and fostered a sense of connection for carer participants. This was in line with recent findings that peer interactions in digital interventions helped to improve emotional well-being and perceived support among informal carers.^42,52,53,74,75^ Participants suggested that including more interactive or synchronous elements could enhance engagement, indicating potential areas for future MOOC improvement.

### Impact of the MOOC on participants’ learning (level 2)

Level 2 of the Kirkpatrick model evaluates what participants learned during the educational programme. In our study, both carer and non-carer participants generally reported an improved understanding of psychosis and its management (e.g., symptom profiles, relapse indicators, treatment options). Carer participants felt that the course addressed longstanding information gaps that had been left unfilled because key details had never been provided to them or because they were not aware of what they needed to know. These findings align with existing literature demonstrating that digital psychoeducation enhances disorder-specific knowledge and understanding^36,47,76,77^ and with guideline-level recommendations to provide structured information to carers for the management of psychosis.^30^

The course’s multimodal design accommodated diverse learning styles. Neurodivergent participants such as autistic or dyslexic individuals reported that the different formats facilitated their engagement and understanding of course material. Evidence suggests that providing learning materials in multiple formats can reduce cognitive load and enhance learning outcomes for neurodivergent individuals.^78^ Additionally, multimodal resources in MOOCs have been shown to increase learner engagement.^79^

Building on the emotional support highlighted in Level 1, talking-head lived-experience videos and discussion forums were felt to have contributed to learning by strengthening carer participants’ confidence in their caring role, knowledge and understanding, and appraisals of self-efficacy. These components, individually and in combination, supported both carers and non-carers to take stock and reflect on their own experiences and learn from others’ perspectives. We acknowledge that applying Kirkpatrick’s model to qualitative data relies on participants’ self-reports rather than objective measures, which may limit interpretive certainty. Carers in our study reported feeling more capable of advocating for relatives and implementing effective strategies. Many also described adopting more hopeful perspectives on both the illness and its implications for themselves, which have previously been linked to reduced burnout^80^ and improved carer wellbeing.^81^

These findings align with literature emphasising the value of experiential knowledge exchange and peer support in psychoeducational interventions, which can enhance knowledge co-construction^36,37,82,83^ and carers’ confidence, preparedness, coping abilities, and reflective understanding of experiences in psychosis^48,84,85^ and other long-term conditions.^86–88^ This is particularly important given that carers frequently report feeling unprepared and lacking confidence,^63,89,90^ as the caring role often occurs without prior preparation time.

Beyond knowledge acquisition, the reported increases in confidence and self-compassion may reflect improved reflective capacity. In this sense, learners became better able to interpret psychosis-related experiences, recognise their own emotional responses, and adopt less self-blaming caregiving positions. This interpretation is consistent with metacognitive models of psychosis,^48^ which emphasise the role of self-reflection, understanding others’ mental states, and integrating illness-related experiences into coherent narratives.

### Impact of the MOOC on participants’ behaviour (level 3)

Level 3 of the Kirkpatrick model evaluates the extent to which participants self-reported application of what they have learned in real-world settings. Participants reported using knowledge from the MOOC to adapt daily routines, encourage relatives’ engagement in activities, monitor medication side effects, and communicate more calmly. Some participants also self-reported improved interactions with healthcare providers and felt better able to influence care decisions. These self-reported applications reflect broader evidence that psychoeducational interventions, including those delivered as MOOCs,^91^ can help carers translate their learning into practical strategies that enhance the care they provide to their relatives.^92^

In addition, echoing evidence on the potential benefits of MOOCs as tools to support professional development in healthcare,^93–95^ several participants – both carers and non-carers – self-reported applying the course to their current professional roles or drawing inspiration for future careers. MOOCs hold particular value in this regard by providing scalable, repeatable learning opportunities that may overcome geographical and financial barriers.^40^ It is arguable, therefore, that MOOCs could represent a sustainable approach to strengthening both carer education and the global health workforce.

### Impact of the MOOC at the system level (level 4)

Level 4 of the Kirkpatrick model considers the broader outcomes and potential systemic impact of an intervention based on participants’ perceived impacts. While our cross-sectional design does not allow for direct measurement of long-term outcomes, participants’ reports point to possible wider benefits. These included perceived improvements in the wellbeing, engagement, and daily functioning of the relatives they cared for, reflecting the possible knock-on effects of applied learning (Level 3). Participants also described perceiving increased confidence in advocacy and more effective communication with healthcare providers, which they felt could improve interactions with services. Additionally, several participants self-reported professional applications, such as informing current roles or guiding future career decisions, suggesting wider perceived personal and professional development impacts. These findings are exploratory and based on participant perceptions rather than objective measures. Future research is needed to examine whether such perceived effects are sustained and translate into measurable outcomes in care practice or service contexts.

### Implications for MOOC design and future development

Taken together, participants’ accounts suggest several practical implications for future MOOC design. Introducing optional live sessions may help enhance interaction and reduce the sense of learning in isolation that can come with fully asynchronous delivery. Some participants also felt that the four-week format was relatively compressed, suggesting that a longer access window or more flexible pacing could support deeper engagement with the material. In addition, clearer signposting between content aimed at carers and that intended for professionals or non-carer learners may improve the experience for this mixed audience. While the course was generally viewed as accessible, participants’ reflections also point to the value of further developing culturally adapted and potentially multilingual content to strengthen inclusivity and relevance across diverse learner groups. Finally, given the emotionally sensitive and open-access nature of the course, strengthening moderation of discussion spaces, safeguarding processes for distress, privacy protections, and clear crisis-support signposting may also help ensure safe engagement alongside learning. These implications are supported by broader evidence on digital interventions in early psychosis, particularly the importance of accessibility, engagement, peer support, and scalable models of care, including telepsychiatry and mobile applications.^35^

### Limitations

Several limitations should be considered when interpreting these findings. First, the study’s cross-sectional design and qualitative self-report measures limits the ability to draw any causal inferences or measure long-term behavioural and systemic outcomes. While participants described applying MOOC knowledge in real-world settings, qualitative health research may be influenced by social desirability bias^96^ or recall bias,^97^ potentially overestimating the intervention’s impact.

Second, the modest sample consisted of self-selected participants who were motivated to seek information online, which may not represent carers with limited digital literacy or access. This reflects a broader challenge in digital health, where individuals with higher digital literacy are more likely to engage with online health interventions.^98^ Research indicates that carers who are older, living in rural or remote areas, or with limited technical skills can face barriers to engaging with MOOCs.^47^ These disparities are exacerbated in global contexts as many MOOCs are delivered in the English language^99^ and may not account for cultural differences, or require reliable internet and digital devices, which may restrict participation in low-resource settings.^100^ While such barriers were not widely reported in our sample, likely reflecting the self-selecting, digitally confident participant sample, they remain critical considerations for scaling MOOCs to more diverse and racially or culturally minoritised carers.

Third, the sample composition may limit the transferability of findings to the broader population of informal carers. Among participants with caring experience, most were supporting a child, which may disproportionately reflect the needs and perspectives of this group. Qualitative caregiving research often highlights how specific subgroups (e.g. older carers, spousal carers) may have distinct experiences,^101^ and findings from studies dominated by one carer type therefore may not fully capture the breadth of informal caregiving contexts. In addition, three participants did not have direct caregiving experience. Although their perspectives were valuable in understanding the wider relevance of the MOOC, their inclusion may limit the extent to which the findings can be applied specifically to informal carers.

Fourth, while later interviews largely reinforced previously identified themes, it is possible that additional interviews may have generated further insights or nuanced understandings of participant experiences.

Fifth, limited demographic data were collected on participants’ ethnic backgrounds. This restricts the ability to explore how experiences of the MOOC may differ across racially and ethnically minoritised groups, particularly given evidence that such groups may face additional barriers to accessing support.^32,68^ Examining these differences would be an important focus for future research but was beyond the scope of the present study.

Finally, as a qualitative study, the absence of objective measures of changes in care recipient and participant outcomes (e.g., carer wellbeing or professional practice) means that Level 3 and Level 4 Kirkpatrick outcomes are based on participant perceptions rather than observed behaviour or system metrics. Further research incorporating longitudinal studies with mixed-methods evaluation would strengthen evidence for the sustained and systemic impact of carer-focused MOOCs, including emerging approaches to digital assessment of metacognitive processes such as ecological momentary assessment and confidence-based self-appraisal.^102^

## Conclusions

This study provides preliminary evidence that a model of online training can offer an accessible, flexible, and supportive learning platform for individuals engaging with caregiving in psychosis and other interested learners. Among a small, self-selected sample of interviewed learners from several countries, the MOOC was perceived as accessible, validating, and practically useful. Subjective reports suggest that participation enhanced understanding of psychosis, increased confidence in caring roles, supported emotional wellbeing, and inspired practical changes in caregiving and professional development. While these findings are primarily based on self-reported experiences and a cross-sectional design, they highlight the potential of MOOCs as scalable tools to address learners’ informational and emotional needs in the context of psychosis. Future research should aim to strengthen the evidence base by examining long-term impacts and identifying ways to ensure equitable access for diverse and underserved learners.

## Supplemental material

Supplemental material - Supplemental material for Learner experiences of a carer-focused massive open online course on psychosis: A qualitative study Supplemental material for Learner experiences of a carer-focused massive open online course on psychosis: A qualitative study by Kalya Win Aung, Angela Kibia, Juliana Onwumere in DIGITAL HEALTH.

Supplemental material - Supplemental material for Learner experiences of a carer-focused massive open online course on psychosis: A qualitative study Supplemental material for Learner experiences of a carer-focused massive open online course on psychosis: A qualitative study by Kalya Win Aung, Angela Kibia, Juliana Onwumere in DIGITAL HEALTH.
