# Supplementary material for: Learner experiences of a carer-focused massive open online course on psychosis: A qualitative study
Source: Digit Health. 2026 Aug 25;12:20552076261470199. doi: 10.1177/20552076261470199 (PMC13519204; doi:10.1177/20552076261470199)
Supplement: Supplementary file 2 [file sj-pdf-2-dhj-10.1177_20552076261470199.pdf]

Supplementary File 2. Full Interview Guide

Level 1: Reaction

How would you describe your experiences of learning on the course?

How would you describe your overall satisfaction with the course content and delivery?

What motivated you to spend time learning on this MOOC?

Level 2: Learning

What areas of the course did you find the most helpful?

What areas of the course did you find the least helpful?

Did you gain any new insights or knowledge from the course? Can you share examples?

In what ways do you think the course has contributed to your understanding of caring for individuals with psychosis and schizophrenia?

Are there specific topics surrounding caring for psychosis that you feel were not adequately covered by the course?

Level 3: Behaviour

Have you been able to apply any concepts or skills learned in the course to your professional or personal life?

Can you describe any changes in your approach or behaviour resulting from the course?

Are there particular strategies or interventions from the course that you find particularly effective in real-world situations?

Can you identify any areas where you feel more confident in your ability to address the needs of individuals with psychosis and schizophrenia?

Level 4: Impact

Have you noticed any positive outcomes or improvements in your work or personal life as a result of participating in the course?

How well has this course helped you to meet your learning goals?

How could the course be improved to help you better meet your objectives?

Please specify any challenges you encountered in taking this MOOC.

Is there anything else that you'd like to share about your experience on the course that we've not yet talked about?
